# Case report: Incidental MALT lymphoma of the left adrenal gland mimicking a metastatic spread within durvalumab maintenance treatment in inoperable stage III non-small cell lung cancer

**DOI:** 10.3389/fonc.2024.1226422

**Published:** 2024-03-19

**Authors:** Lukas Käsmann, Esra Degerli, Karim El-Marouk, Farkhad Manapov

**Affiliations:** ^1^ Department of Radiation Oncology, University Hospital, Ludwig-Maximilians-University (LMU) Munich, Munich, Germany; ^2^ German Center for Lung Research (DZL), Partner Site Munich, Munich, Germany; ^3^ German Cancer Consortium (DKTK), Partner Site Munich, Munich, Germany; ^4^ Private Practise ´Die RADIOLOGIE´, Munich, Germany

**Keywords:** PET/CT, NSCLC, durvalumab, MALT lymphoma, immune-related side effect

## Abstract

Durvalumab after chemotherapy in non-operable stage III non-small cell lung cancer (NSCLC) is the standard of care worldwide. We present a patient with the incidental discovery of a unilateral MALT lymphoma of the adrenal gland and adrenalitis during durvalumab maintenance treatment detected by 18F-FDG-PET/CT. We assessed the clinical and histopathological findings, radiological examinations and overall treatment. Our work emphasizes the significance of considering other differential diagnoses and the importance of multidisciplinary treatment of the findings, especially within clinical trials.

## 1 Background

Immunotherapy plays a vital role in the treatment of lung cancer today. However, in some cases, immunotherapy-related adverse events (irAEs) develop during this therapy. They can be diagnosed in positron emission tomography–computed tomography (PET/CT) and may occur in every organ system, such as skin, gastrointestinal tract, endocrine system, respiratory system (1). Adverse events during immunotherapy do not always have to be related to it. Our patient had both irAE and MALT lymphoma of the adrenal gland, which was not likely caused by the therapy.

MALT lymphoma is a subtype of non-Hodgkin lymphoma and usually manifests extranodal in the gastrointestinal tract (2) but can also occur in other organ systems (3), for example the adrenal gland (4, 5). However, misdiagnosis may occur due to the low incidence (6). The adrenal gland is a common site for metastasis from lung cancer and enlargement of the adrenal gland can be misinterpreted as a metastasis (7, 8). Some possible differential diagnoses of an adrenal mass include adenoma, myelolipoma, cyst, lipoma, pheochromocytoma, primary adrenal cancer, hyperplasia, and tuberculosis (9).

We present a patient with the incidental discovery of a unilateral MALT lymphoma of the adrenal gland and adrenalitis during immunotherapy with durvalumab after chemoradiotherapy (CRT) in stage III non-small cell lung cancer (NSCLC) detected by 18F-FDG-PET/CT. We assessed the clinical as well as intraoperative findings, radiological examinations, and overall treatment.

## 2 Case presentation

### 2.1 Diagnosis and treatment

**Figure 1:**
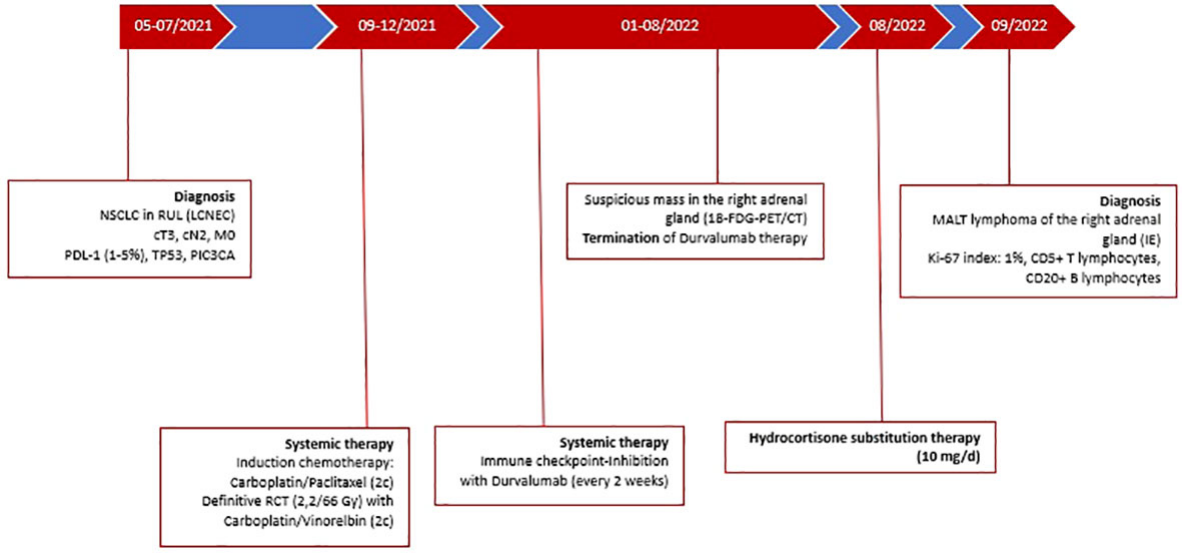
Timeline of patients’ treatment and follow-up.

We present the case of a 67-year-old Caucasian female patient who was referred to our department in May 2021 (**Figure 1**). The patient initially presented with progressive dyspnea and cough. A subsequent computed tomography (CT) scan revealed a suspicious malignant mass in the upper lobe of the right lung and was clinically diagnosed as locally advanced NSCLC. Histological evaluation and next-generation sequencing revealed a large cell neuroendocrine lung carcinoma (LCNEC) with S37C mutation in the CTNNB1 gene, E542 mutation in the PIK3CA gene, and inactivating mutation in the TP53 gene. Nuclear staining for Ki-67 was performed using anti-Ki67 antibody (MIB-1, 1:100, Dako). Ki-67 expression was scored as 50% evaluated semiquantitatively as a percentage of positive cells upon manual counting of 500 to 1,000 tumor cells. For the detection of programmed death-ligand 1 (PD-L1), prediluted PD-L1 rabbit monoclonal antibody (SP263; Ventana Medical Systems, Oro Valley, Arizona) was used and measured as 5% using the tumor proportion score (TPS). Both stainings were performed on a Ventana Benchmark Ultra autostainer using the ultraView diaminobenzidine kit (Ventana Medical Systems, Oro Valley, AZ).

Initial positron emission tomography/computed tomography (18-FDG-PET/CT) staging and cranial magnetic resonance imaging (cMRI) in July/August 2021 showed metastases in the hilar lymph nodes on the right side, which resulted in stage IIIB (cT3, cN2, M0) according to UICC TNM staging (8th edition) (10).

Relevant medical history findings included active smoking (50 p/y), breast cancer (diagnosed in 2006, non-active), peripheral arterial disease, and chemotherapy-associated pancytopenia CTCAE II.

Beginning in September 2021, our patient underwent induction chemotherapy with two cycles of carboplatin (AUC5) and paclitaxel (150 mg/m²). After local response, concurrent CRT (30 fractions × 2.2 Gy/66 Gy) with two cycles of concurrent platinum-based chemotherapy was administered. The patient received carboplatin (AUC5 i.v.) on day 1 of each cycle and 25 mg/m^2^ vinorelbine i.v. on day 1 and day 8 of each cycle.

The patient was treated with immunotherapy with PD-L1 antibody durvalumab according to the phase III PACIFIC trial (NCT02125461) (11) starting in January 2022. Immunotherapy was given every 2 weeks. During durvalumab therapy, the patient’s general condition deteriorated in August 2022 with a diagnosis of Addison’s disease due to CTCAE grade II autoimmune adrenalitis, which was potentially immune-mediated. This led to the discontinuation of durvalumab therapy.

A further 18F-FDG PET/CT scan revealed increased metabolic activity in the right adrenal gland (SUVmax: 6.7) with suspicion of possible metastasis. After multidisciplinary discussions, a laparoscopic adrenalectomy was performed in August 2022. Histological examination of the resected soft tissue (5.5 × 4.3 × 0.4 cm) revealed an enlarged adrenal gland with a maximum diameter of 3.3 cm. In fact, the patient had histomorphologically highly atypical adrenal tissue; the medullary zone was distinguishable, and the adrenal cortex tissue was completely depleted. Using conventional light microscopy, there were neither cells of the zona glomerulosa, nor zona fasciculata or zona reticularis. Instead, immunohistological evaluation showed a band-like lympho-plasmacytoid infiltration/inflammation with a dominant diffuse and focally nodular infiltration of CD5-positive T-lymphocytes and partially grouped CD20-positive B-lymphocytes. Numerous CD38-positive plasma cells were detected with a dominance of lambda over kappa-positive plasma cells. Further molecular pathological examination detected a clonality of the IgH gene but no mutation in the MyD88 gene. In summary, an indolent non-Hodgkin’s lymphoma of the B-cell lineage was found in the right adrenal gland, suggesting a MALT lymphoma. Further endoscopic diagnostic by bioptic securing showed no evidence of other manifestations of the MALT-Lymphoma (e.g., gastrointestinal tract). A bone marrow puncture was rejected by the patient.

### 2.2 Outcome and follow-up

In close cooperation with our endocrinology department, a substitution therapy with *Hydrocortisone* 10 mg/day was initiated. Continuation of the immunotherapy with durvalumab was not planned according to the decision of the tumor board. However, adjuvant therapy for the MALT-Lymphoma was not indicated. A follow-up 18F-FDG-PET/CT performed in January 2023 showed a stable disease situation with no local progression of the NSCLC, no metastatic lesions, and no increased metabolism indicating a possible local recurrence in the field of the resected adrenal gland.

## 3 Discussion

This case report demonstrates the importance of histological evaluation and differential diagnoses, especially in patients with complex patient history.

Immunotherapy, particularly the use of checkpoint inhibitors (ICIs), has revolutionized treatment of NSCLC in recent years (12). While ICIs have shown remarkable efficacy in some patients, they can also lead to irAEs, which can range from mild to severe and life-threatening (13). IrAEs can affect any organ system and can present with a wide range of symptoms, including skin rash, colitis, hepatitis, pneumonitis, and endocrinopathies (14–16). The incidence and severity of irAEs depend on the type of ICIs, the dose, and the duration of treatment (17).

The meta-analysis by Wang et al. evaluated 46 trials with a total of 12,808 patients treated with PD-1/PD-L1 inhibitors. In this analysis, the patients received treatment with nivolumab (21 studies, 45.65%), pembrolizumab (17 studies, 36.96%), atezolizumab (5 studies, 10.87%), durvalumab (one study, 2.17%), and avelumab (one study, 2.17%). On average, the incidence of irAE of any grade was 32.92% in every organ system. the following specific incidences of irAEs were determined after subdividing into the individual organ systems: skin (13%), gastrointestinal tract (10%–13%), respiratory system (3%), endocrine system (≤2%), liver (≤2%), and kidney (<1%) (1).

Early diagnosis and management of irAEs are crucial for improving patient outcomes (18). One diagnostic tool that has shown promise in detecting irAEs is 18F-FDG-PET/CT (19), which differentiates them from other disease processes such as infection or cancer progression (20).

In addition to detecting irAEs, 18F-FDG-PET/CT can also be used to predict the development of irAEs. A study by Nobashi et al. evaluated the use of 18F-FDG-PET/CT in predicting the possible outcomes of irAE in patients receiving ICIs. The study included 40 patients who underwent 18F-FDG-PET/CT before and after the ICI therapy. Patients with irAE that could be detected by 18F-FDG-PET/CT were significantly associated with a favorable long-term outcome (P = 0.002) (21).

In conclusion, 18F-FDG-PET/CT is a promising tool for the diagnosis and monitoring of irAEs in patients undergoing immunotherapy. While further research is needed to fully evaluate the utility of 18F-FDG-PET/CT in this context, existing studies suggest that 18F-FDG-PET/CT can provide valuable information for the early detection and management of irAEs.

18F-FDG-PET/CT is also a well-established imaging method for detecting, staging, and monitoring various cancer entities. The accuracy of 18F-FDG-PET/CT in cancer patients depends on several factors, such as the tumor size, location, type, and stage of disease. In recent studies, 18F-FDG-PET/CT has demonstrated good sensitivity and specificity for detecting different types of primary cancer and distant metastasis, including lung cancer and adrenal masses. A meta-analysis about the diagnostic accuracy of 18F-FDG-PET/CT for the characterization of adrenal masses performed by Kim et al. showed in 29 studies with 2,421 patients in total a sensitivity of 91% [95% CI (0.88–0.94)] and specificity of 91% [95% CI (0.87–0.93)] (22).

Ma et al. displayed that SUVmax of malignant lesions (10.0 ± 5.8) was higher than that of benign lesions (5.4 ± 5.3, P < 0.05), and as a result of this finding, the SUV-ROC method has a sensitivity of 81.25% and specificity of 72.91% (23). Another study by Perry et al. demonstrated a higher sensitive accuracy of 18F-FDG-PET/CT in detection of non-gastric MALT-Lymphoma versus gastric MALT-Lymphoma (75% vs. 38.9%) (24). Despite its limitations, 18F-FDG PET/CT remains a respected method for diagnosing and monitoring suspicious tumors of the adrenal gland.

In our patient, the increased metabolic activity in the right adrenal gland seen on 18F-FDG-PET/CT raised suspicion of an adrenal metastasis of NSCLC. However, histologic examination revealed a MALT lymphoma. This emphasizes the importance of histological examination of suspected metastases. A systematic review of adrenal biopsy by Bancos et al. using 32 studies found that adrenal biopsy had a sensitivity of 87% [95% CI (0.78–0.93)] and a specificity of 100% [95% CI (0.7–1.00)] for the overall diagnosis of malignancy. For the diagnosis of metastases, the sensitivity was 87% [95% CI (0.74–0.94)] and the specificity was 96% [95% CI (0.89–0.98)] (25). Stone et al. created a diagnostic algorithm that only integrates biopsy for histopathological investigation in suspected benign adenoma with SUV max < 3.1 or SUVmax > 3.1 and SUV ratio < 2.5 in PET/CT (26). Otherwise, malignant metastasis is suspected and adrenalectomy is indicated with therapeutic and diagnostic benefit by performing ex vivo histologic examinations. As stated, SUVmax analysis in PET/CT gives information whether the adenoma is benign or malignant, but the comparison of the histological finding leads to the final diagnosis.

Retroperitoneal supra-renal masses are often diagnosed coincidentally in the context of imaging of the abdomen and are then classified as incidentaloma. The origin of these suspect adrenal masses must be evaluated to discuss the following treatment, which differs if it is an adrenal (pheochromocytoma, adrenal hyperplasia, cortical adenoma) or extra-adrenal (malignancies, infections, hemorrhage) origin. As Frey et al. state in their review, 3.5% of the adrenalectomies are mistakenly performed under the indication of an adrenal disease (27). However, most incidentalomas are metastasis of other tumorous sites. Also, rare manifestations such as ganglioneuroma, hemangioendothelioma, and hemangioma must be considered. In 2016, the European Society of Endocrinology published a Clinical Practice Guideline for the management of such findings in the adrenal gland (28).

There are few clinical trials addressing PD-L1-targeted therapy with durvalumab in hematologic malignancies such as MALT-Lymphoma. The Phase 1/2 FUSION NHL 001 study by Casulo et al. aims to evaluate the security and efficacy of durvalumab as monotherapy and in combination with other full agents in patients with lymphoma (29). They found that durvalumab monotherapy and durvalumab combinations did not provide additional benefit while adding the treatment-related toxicity of PD‐L1 blockade.

In summary, this case demonstrates the importance of multidisciplinary discussion in the management of complex cases, especially when rare histologic subtypes are given and within clinical trials.

## 4 Conclusion

Despite its side effects, immunotherapy is an established component in the treatment of lung cancer. 18F-FDG-PET/CT plays a crucial role in diagnosis and during treatment to detect complications early. Nevertheless, if malignant processes are suspected, histologic confirmation must be performed. Otherwise, unexpected differential diagnoses may be misinterpreted. We emphasized the significance of considering other differential diagnoses in these patients and the importance of multidisciplinary treatment of the findings, especially within clinical trials.

## Ethics statement

Written informed consent was obtained from the participant/patient(s) for the publication of this case report.

## Author contributions

LK, ED and KE-M wrote the manuscript. FM supervised the writing. All authors contributed to the article and approved the submitted version.

## References

[1] Wang PF Chen Y Song SY Wang TJ Ji WJ Li SW. Immune-related adverse events associated with anti-PD-1/PD-L1 treatment for Malignancies: A meta-analysis. Front Pharmacol. (2017) 8:730. doi: 10.3389/fphar.2017.00730 29093678 PMC5651530

[2] Ghimire P Wu GY Zhu L. Primary gastrointestinal lymphoma. World J Gastroenterol. (2011) 17:697–707. doi: 10.3748/wjg.v17.i6.697 21390139 PMC3042647

[3] Harris GJ Tio FO Von Hoff DD. Primary adrenal lymphoma. Cancer. (1989) 63:799–803. doi: 10.1002/1097-0142(19890215)63:4<799::AID-CNCR2820630432>3.0.CO;2-5 2644013

[4] Di Rocco A Petrucci L Assanto GM Martelli M Pulsoni A. Extranodal marginal zone lymphoma: pathogenesis, diagnosis and treatment. Cancers (Basel). (2022) 14:1742. doi: 10.3390/cancers14071742 35406516 PMC8997163

[5] Moreau L Gobet F Grise P Perraudin V Lefebvre H. Marginal-zone lymphoma mimicking adrenal myelolipoma on computed tomography scan. J Clin Endocrinol Metab. (2010) 95:4173–4. doi: 10.1210/jc.2010-0329 20823468

[6] Wang Y Ren Y Ma L Li J Zhu Y Zhao L. Clinical features of 50 patients with primary adrenal lymphoma. Front Endocrinol. (2020) 11:595. doi: 10.3389/fendo.2020.00595 PMC7541938 33071959

[7] Glomset DA. The incidence of metastasis of Malignant tumors to the adrenals. Am J Cancer. (1938) 32:57–61. doi: 10.1158/ajc.1938.57

[8] Libè R Dall’Asta C Barbetta L Baccarelli A Beck-Peccoz P Ambrosi B. Long-term follow-up study of patients with adrenal incidentalomas. Eur J Endocrinol. (2002) 147:489–94. doi: 10.1530/eje.0.1470489 12370111

[9] Arnold DT Reed JB Burt K. Evaluation and management of the incidental adrenal mass. Proc (Bayl Univ Med Cent). (2003) 16:7–12. doi: 10.1080/08998280.2003.11927882 16278716 PMC1200803

[10] Brierley JD Gospodarowicz MK Wittekind C. TNM Classification of Malignant Tumours. 8th Edition. Wiley-Blackwell, Hoboken, New Jersey (United States): Wiley (2023). Available at: https://www.wiley.com/en-es/TNM+Classification+of+Malignant+Tumours%2C+8th+Edition-p-9781119263579.

[11] Antonia SJ Villegas A Daniel D Vicente D Murakami S Hui R. Durvalumab after chemoradiotherapy in stage III non–small-cell lung cancer. New Engl J Med. (2017) 377:1919–29. doi: 10.1056/NEJMoa1709937 28885881

[12] Reck M Rodríguez-Abreu D Robinson AG Hui R Csőszi T Fülöp A. Updated analysis of KEYNOTE-024: pembrolizumab versus platinum-based chemotherapy for advanced non-small-cell lung cancer with PD-L1 tumor proportion score of 50% or greater. J Clin Oncol. (2019) 37:537–46. doi: 10.1200/JCO.18.00149 30620668

[13] Larkin J Chiarion-Sileni V Gonzalez R Grob JJ Rutkowski P Lao CD. Five-year survival with combined nivolumab and ipilimumab in advanced melanoma. N Engl J Med. (2019) 381:1535–46. doi: 10.1056/NEJMoa1910836 31562797

[14] Grover S Rahma OE Hashemi N Lim RM. Gastrointestinal and hepatic toxicities of checkpoint inhibitors: algorithms for management. Am Soc Clin Oncol Educ Book. (2018) 38:13–9. doi: 10.1200/EDBK_100013 30231401

[15] Joshi MN Whitelaw BC Palomar MTP Wu Y Carroll PV. Immune checkpoint inhibitor-related hypophysitis and endocrine dysfunction: clinical review. Clin Endocrinol (Oxf). (2016) 85:331–9. doi: 10.1111/cen.13063 26998595

[16] Chan KK Bass AR. Autoimmune complications of immunotherapy: pathophysiology and management. BMJ. (2020) 369:m736. doi: 10.1136/bmj.m736 32253223

[17] Martins F Sofiya L Sykiotis GP Lamine F Maillard M Fraga M. Adverse effects of immune-checkpoint inhibitors: epidemiology, management and surveillance. Nat Rev Clin Oncol. (2019) 16:563–80. doi: 10.1038/s41571-019-0218-0 31092901

[18] Özdemir BC Espinosa da Silva C Arangalage D Monney P Guler SA Huynh-Do U. Multidisciplinary recommendations for essential baseline functional and laboratory tests to facilitate early diagnosis and management of immune-related adverse events among cancer patients. Cancer Immunol Immunother. (2023) 72(7):1991–2001. doi: 10.1007/s00262-023-03436-0 PMC10264466 37017694

[19] Wong ANM McArthur GA Hofman MS Hicks RJ. The advantages and challenges of using FDG PET/CT for response assessment in melanoma in the era of targeted agents and immunotherapy. Eur J Nucl Med Mol Imaging. (2017) 44:67–77. doi: 10.1007/s00259-017-3691-7 28389693

[20] Cho SY Huff DT Jeraj R Albertini MR. FDG PET/CT for assessment of immune therapy: opportunities and understanding pitfalls. Semin Nucl Med. (2020) 50:518–31. doi: 10.1053/j.semnuclmed.2020.06.001 PMC8201415 33059821

[21] Nobashi T Baratto L Reddy SA Srinivas S Toriihara A Hatami N. Predicting response to immunotherapy by evaluating tumors, lymphoid cell-rich organs, and immune-related adverse events using FDG-PET/CT. Clin Nucl Med. (2019) 44:e272–9. doi: 10.1097/RLU.0000000000002453 30688730

[22] Kim SJ Lee SW Pak K Kim IJ Kim K. Diagnostic accuracy of 18F-FDG PET or PET/CT for the characterization of adrenal masses: a systematic review and meta-analysis. Br J Radiol. (2018) 91:20170520. doi: 10.1259/bjr.20170520 29327944 PMC6223272

[23] Ma G Zhang X Wang M Xu X Xu B Guan Z. Role of 18F-FDG PET/CT in the differential diagnosis of primary benign and Malignant unilateral adrenal tumors. Quant Imaging Med Surg. (2021) 11:2013–8. doi: 10.21037/qims-20-875 PMC8047365 33936982

[24] Perry C Herishanu Y Metzer U Bairey O Ruchlemer R Trejo L. Diagnostic accuracy of PET/CT in patients with extranodal marginal zone MALT lymphoma. Eur J Haematol. (2007) 79:205–9. doi: 10.1111/j.1600-0609.2007.00895.x 17662066

[25] Bancos I Tamhane S Shah M Delivanis DA Alahdab F Arlt W. DIAGNOSIS OF ENDOCRINE DISEASE: The diagnostic performance of adrenal biopsy: a systematic review and meta-analysis. Eur J Endocrinol. (2016) 175:R65–80. doi: 10.1530/EJE-16-0297 27257146

[26] Stone WZ Wymer DC Canales BK. Fluorodeoxyglucose-positron-emission tomography/computed tomography imaging for adrenal masses in patients with lung cancer: review and diagnostic algorithm. J Endourol. (2014) 28:104–11. doi: 10.1089/end.2013.0380 PMC3880901 23927734

[27] Frey S Caillard C Toulgoat F Drui D Hamy A Mirallié É. Non-adrenal tumors of the adrenal area; what are the pitfalls? J Visceral Surg. (2020) 157:217–30. doi: 10.1016/j.jviscsurg.2020.02.004 32201083

[28] Fassnacht M Arlt W Bancos I Dralle H Newell-Price J Sahdev A. Management of adrenal incidentalomas: European Society of Endocrinology Clinical Practice Guideline in collaboration with the European Network for the Study of Adrenal Tumors. Eur J Endocrinol. (2016) 175:G1–34. doi: 10.1530/EJE-16-0467 27390021

[29] Casulo C Santoro A Cartron G Ando K Munoz J Le Gouill S. Durvalumab as monotherapy and in combination therapy in patients with lymphoma or chronic lymphocytic leukemia: The FUSION NHL 001 trial. Cancer Rep (Hoboken). (2023) 6:e1662. doi: 10.1002/cnr2.1662 35852004 PMC9875673

